# Translation, Adaptation, and Validation of the Portuguese Version of the Exercise of Self-Care Agency Scale

**DOI:** 10.3390/healthcare12020159

**Published:** 2024-01-10

**Authors:** Fátima Cunha, Maria do Rosário Pinto, Susan Riesch, Pedro Lucas, Sofia Almeida, Margarida Vieira

**Affiliations:** 1 Interdisciplinary Research Center in Health (CIIS), 4169-005 Porto, Portugal; ssalmeida@ucp.pt (S.A.); mmvieira@ucp.pt (M.V.); 2 Life Quality Research Center (CIEQV), 2040-413 Rio Maior, Portugal; 3 Higher School of Health, Santarém Polytechnic University, 2005-075 Santarém, Portugal; 4 Health Sciences Research Unit: Nursing (UICISA-E), 3046-851 Coimbra, Portugal; mrosario.pinto@esel.pt; 5 Nursing Research, Innovation and Development Center in Lisbon (CIDNUR), Nursing School of Lisbon, 1600-190 Lisbon, Portugal; prlucas@esel.pt; 6 School of Nursing, University of Wisconsin-Madison, Madison, WI 53705, USA; 10usplayer@gmail.com; 7 Faculty of Health Sciences and Nursing (FCSE), Catholic University of Portugal, 4169-005 Porto, Portugal

**Keywords:** nursing, older adults, psychometric properties, self-care, self-care agency

## Abstract

Assessing a person’s capacity to engage in self-care behaviours is another added value in identifying one’s potential to care for oneself in the health domain that contributes to planning person-centred care. This study aimed to translate, adapt, and validate the Exercise of Self-Care Agency (ESCA) Scale by Kearney and Fleischer, revised by Riesch and Hauck for the Portuguese version, using a sample of 625 elderly people living at home in Portugal. A cross-cultural adaptation process follows the stages of translation, synthesis, back-translation, and consensual solution for the translation process and pretesting. Construct validity was tested using exploratory factor analysis, and factor structure was subjected to confirmatory factor analysis. Reliability was determined by analysing internal consistency, resorting to Cronbach’s alpha coefficient. This resulted in an instrument formed of 29 items, keeping the factor structure conceptually aligned with the underlying theory. Cronbach’s alpha coefficient values were 0.87 for the global scale and varied between 0.65 and 0.84 for the subscales. The final four-factor model showed an acceptable quality of fit. The Portuguese version of the ESCA shows appropriate validity and reliability for use in future research and health contexts.

## 1. Introduction

People’s capacity to care for themselves has been promoted further in the health domain, emphasising personal responsibility and the potential and active role of each person in promoting their own health and well-being. The World Health Organisation (WHO) defines self-care as the capacity of individuals, families, and communities to promote health, prevent illness, maintain health, and cope with illness and disability with or without the support of a healthcare worker [1] (p. ix).

The scope of self-care expressed in this concept integrates health promotion; disease prevention and control; self-medication; giving care to dependent people; seeking hospital, specialist or primary care when needed; and rehabilitation, including palliative care [2]. This scope reveals the enormous impact that the promotion of self-care has on the health systems of all countries, as well as the importance that personal responsibility, autonomy, and self-empowerment assume in individual health and well-being.

Highlighting the importance of this issue, the WHO emphasises that the difficulty of access to health services, the shortage of health professionals, and global threats, including humanitarian crises and pandemics such as COVID-19, are associated with an urgent need to find innovative strategies, such as those proposed in the guideline on self-care interventions for health and well-being [2]. This perspective is shared by the International Center for Self-Care Research, an organisation with a mission to disseminate and prioritise self-care as the first line of approach in healthcare, and by the International Self-Care Foundation, an organisation that promotes healthy lifestyles through educational support and information on self-care and healthy lifestyles [3].

Accordingly, due to its importance, self-care is a multidisciplinary concept (medicine, psychology, pharmacy, or nursing), expressed in creating and utilising different models, theories, and frameworks [3,4]. In the context of this study, models of particularly high pertinence are those that improve self-care capacities and capabilities along with promoting self-care knowledge, namely the model of self-care for health promotion in ageing [5], the model for the self-care of home-dwelling elderly [6], and Orem’s self-care deficit nursing theory [7].

Orem’s self-care deficit nursing theory emerged as one of the first works related to self-care, and it has been mobilised in different studies over time. As a central concept of the theory, self-care is described as a continuous process of actions produced by or for people from birth to death to maintain health and well-being and promote one’s development [7]. It is influenced by basic conditioning factors (age, sex, state of development, state of health, socio-cultural orientation, factors associated with the health system, factors of the family system, patterns of life, environmental factors, and the suitability and availability of resources). However, as a behaviour, it reflects individual styles and specific adaptations to current circumstances and future perspectives [7,8].

Another relevant conceptual element in self-care theory associated with people’s potential to look after themselves and stay healthy is the concept of self-care agency. This concept focuses on the human power activated and shown by a person when they investigate, judge, make decisions, and produce self-care operations (estimative, transitional, and productive types) [8].

There is a self-care deficit when people’s self-care actions do not respond to real or potential therapeutic self-care needs. In this case, nursing interventions are necessary to help satisfy their requirements. When nurses focus on assessing someone’s power and capacity to act to develop self-care, they may guide care from that person’s perspective and develop strategies to promote the internal resources each person uses to look after themselves based on that evaluation. Assessing this capacity is very important, so it is pertinent to have instruments that allow for such evaluation.

Different authors [9,10,11] have identified instruments that assess adult and elderly people’s capacity for self-care, but none of these studies focus on Portugal. Therefore, we selected the Exercise of Self-Care Agency (ESCA) Scale among the identified instruments as it consolidates the underlying theoretical construct and considers domains listed as relevant by the International Self-Care Foundation. This instrument was developed by Kearney and Fleischer in 1979 in the United States of America [12], based on the work conducted by Dorothea Orem and the Nursing Development Conference Group. It assesses a person’s capacity to engage in self-care actions based on four domains: self-worth, motivation, knowledge, and passive response to situations. It has been translated and validated in different countries, including France [13], Japan [14], Turkey [15], and China (Cantonese version) [16]. Overall, the instrument demonstrates validity and appropriate reliability but with different behaviours in different contexts.

We shift our attention to the construct analysis and discriminant validation of the ESCA carried out by Riesch and Hauck [17]. The instrument from this analysis, which we explain below, is conceptually identical, having a few items; thus, its availability in Portuguese language and culture is a gain in providing patient-centred healthcare.

This paper aims to present the translation, adaptation, and validation of the ESCA for the Portuguese language and culture.

## 2. Materials and Methods

### 2.1. Design

This methodological study was developed in two interconnected phases: (1) the translation and cross-cultural adaptation of the instrument and (2) the evaluation of the psychometric properties of the Portuguese version of the scale.

### 2.2. Sample and Data Collection Procedures

A convenience sample of participants who satisfied the following criteria was used: (a) aged 65 or over; (b) living at home; (c) agreeing to voluntarily participate in this study; (d) not presenting mental conditions that would prevent understanding the contents of the questionnaire (defined by the mini-mental state examination—MMSE). Individuals were recruited from the researcher’s contact network using the snowball sampling method. The sample size complies with the estimation principle of the “Rule of Thumb”, in which to analyse K variables (K > 15), and to achieve the proposed adequacy of the sample to carry out the exploratory factorial analysis (EFA)—in the classification of very good (which proposes N = 500)—the minimum sample size must be N = 5K (in this study, 5 × 35 = 175 is the minimum number of participants) [18,19].

The questionnaire is presented in this paper, and data were gathered between September and November 2019. The researcher was always present in applying the MMSE to form the sample, in the procedures regarding informed consent, clarifying the language used in the questionnaire and/or helping participants who could not read.

The sample comprised 625 elderly people aged between 65 and 97, with an average age of 75.18 (SD = 6.82). The age group of 65 to 74 represents 49% of the participants, 40.8% for 75 to 84, and 10.2% for ≥85. The majority (67.2%) are female, and 32.8% are male. Concerning residence, 75.7% say they live with a partner, family or significant other, and 24.3% live alone. As for education, the majority attended or completed primary education (58.7%), and fewer completed higher education (7.4%) or did not attend school (3.7%) (see Table 1).

Describing their state of health, the majority feel they can perform usual self-care activities despite illness (55.5%); 35.5% say they feel healthy; and 9% say they are unable to perform usual self-care activities, needing the help of another/others.

### 2.3. The Instrument Studied

The revised version of Riesch and Hauck [17] of the ESCA is an instrument formed of 35 items, distributed over 4 domains: self-concept, initiative and self-responsibility, knowledge and information seeking, and passivity, which validate the works of Kearney and Fleischer [12]. Each item oriented positively towards self-care was scored from 0 to 4, according to the participant’s response on a 5-point Likert-type scale. A score of 0 corresponded to choosing “Very unlike me”, and a score of 4 corresponded to “Very like me”. Items 3, 6, 8, 13, 18, 21, 24, and 29 are worded negatively regarding the capacity to engage in self-care actions, so the scoring was reversed. A higher score indicated a high capacity to perform self-care actions.

The data for construct analysis and discriminant validity were obtained from three studies that used the ESCA scale (*n* = 506). Sample A was made up of women who were between 32 and 40 weeks pregnant (*n* = 100), Sample B of healthy adolescents (*n* = 110), and Sample C of university lecturers, non-teaching staff, and students (*n* = 296).

The self-concept domain includes items 2, 4, 7, 9, 17, 22, 27, 28, 29, 30, 33, and 35, with coefficients varying between 0.40 and 0.63. These items include terms associated with self-esteem, self-worth, and self-confidence. The initiative and self-responsibility domain includes items 1, 5, 10, 11, 12, 14, 15, 16, 19, 23, 25, and 34, connected with an attitude to act on one’s own initiative and answer for one’s own actions. The coefficients varied between 0.40 and 0.62. Items 18, 20, 26, 31, and 32 include the knowledge- and information-seeking domain, with coefficients ranging from 0.53 to 0.67. Items 3, 6, 8, 13, 21, and 24 make up the passivity domain, conveying the idea that a self-care agent is aware of their self-care resources but chooses not to activate them. The internal consistency of the total scale was α = 0.90 (self-concept α = 0.81, initiative and self-responsibility α = 0.81, knowledge and information seeking α = 0.76, and passivity α = 0.73). The scale had already been used on an elderly population, with the alphas for the total scale and the sub-scales varying between 0.70 and 0.89 [20].

### 2.4. Cross-Cultural Adaptation Process

The translation, cultural adaptation, and psychometric validation of the instrument were carried out according to the guidelines for healthcare research [21,22] aimed for the instrument to keep its semantic, idiomatic, conceptual, and psychometric equivalence.

Translation from English to Portuguese was performed by two bilingual translators whose mother tongue is Portuguese. One of the translators had previous experience translating technical texts and knew about the aim of the instrument and the underlying construct. The other translator had no prior knowledge of the aims of the instrument and had previous experience translating instruments. The two translations were compared, discrepancies were analysed by the researchers in a meeting with the translators, and a consensual version was reached. Independent back-translation was performed by a bilingual translator with the same first language as the author of the original instrument who did not know the study objectives, the original version of the instrument, or contact with the work carried out by the other translators. The next step was to reach a consensual solution for the translation process (considering the original version, translation, and back-translation), calling on experts in the area and a bilingual translator specialising in translating instruments. Minimal discrepancies in meaning were identified in two questions, producing a translation with no relevant semantical or conceptual differences after reflection; therefore, no meaning about the items was lost in this process.

### 2.5. Pre-Test

A pre-test of the translated version was carried out on 45 elderly people with similar characteristics to the target population, having different levels of education. The obtained results revealed a suitable understanding of the instructions to answer on the scale and the items. The response options were frequently mentioned as making little sense in relation to some items, making selection difficult. The physical aspect of the instrument also caused difficulty, particularly marking on the text caused by the item response options, making it awkward to complete and potentially leading to mistakes by the respondents. This analysis resulted in changing the response options on the 5-point Likert scale, which became “Completely agree”, “Agree”, “Neither agree nor disagree”, “Disagree”, and “Completely disagree”, maintaining the score assigned by the scale’s author. Thus, each positively oriented item for self-care was scored from 0 to 4, according to the participant`s response on a 5-point Likert-type scale. A score of 0 corresponded to the choice “Completely disagree” and a score of 4 to “Completely agree”. Scores for items written negatively concerning the ability to perform self-care actions were similarly reversed.

Aware of the relevance of keeping the graphical aspect while simultaneously wanting minimal errors and easy completion, we decided to change the graphical aspect of the Likert scale, which was now represented by a continuous line, which included the abbreviated points of response. These changes were found to be appropriate in subsequent pilot applications since it was easier to answer the items. There were no comments on the displayed text area.

### 2.6. Statistical Procedures

The statistical programs IBM-SPSS Statistics version 27.0 and AMOS (IBM Corp., Armonk, NY, USA) were used to carry out all the analyses.

A descriptive and comparative analysis of the variables was carried out. Continuous variables were described using mean and standard deviation, while categorical variables were described using relative and absolute frequencies.

The exploratory factor analysis technique was applied to identify whether the factor structure of the original scale remained identical when applied to a different population. The sample corresponded to all the items in the scale, so Little’s MCAR test was not carried out.

The main components were extracted using the Varimax rotation method to carry out the EFA. Two tests were used to analyse the suitability of the data for exploratory factor analysis: the Kaiser–Meyer–Olkin (KMO) test, the value of which must be greater than 0.50, and Bartlett’s test of sphericity, which should have a significance value less than 0.05. Both indicate the suitability of the data for factor analysis [23,24].

For the factor analysis, we kept the items with a factor load greater than 0.40 [23]. We used the scree plot and the Kaiser criteria to extract the factors. The total variance explained by the results was analysed.

We determined the internal consistency and reliability of the instrument using Cronbach’s alpha coefficient and composite reliability. These values can vary from 0 to 1, with 0.70 being the minimum value for acceptable reliability [25].

To assess the quality of the fit of the model obtained through the EFA, a confirmatory analysis (CFA) was carried out using AMOS software (version 26.0). The CFA was carried out using the maximum likelihood method, which assumes the independence of the observations, multivariate normality, and the absence of outliers. The normal distribution of the variables was analysed using the asymmetry (Sk) and kurtosis (Ku) coefficients. Outliers were assessed by the square of the Mahalanobis square distance (D2) [25].

To assess the overall fit of the model in the CFA, the following indices were used: the goodness of fit index (GFI) and the comparative fit index (CFI), where a score greater than 0.90 reveals a good fit [25]; the root mean square error of approximation (RMSEA), with an acceptable score between 0.05 and 0.08; and the standardised root mean square (SRMR), with an acceptable score of less than 0.08 [26]. The model with the lowest expected cross-validation index (M)ECVI represents the best fit.

For this purpose, we used the modification indices provided in AMOS and theoretical considerations [25]. The Mardia coefficient was not used because this significance test alone is not a practical assessment of normality, especially in a structural equation model [27].

The structural validity of the scale was tested first with EFA and then with structural equation models. CFA was tested using factorial and convergent validity. Once multivariate normality was confirmed, we tested factorial validity with maximum likelihood.

The proposed factor model is considered valid when all items have a factor loading greater than 0.40 [25,26]. Construct validity was calculated using convergent validity (using the average variance extracted (AVE) for each factor for each factor and considering 0.50 as the minimum value) and discriminant validity, confirmed by the confirmed by evidence that the AVE for each pair of factors is equal to or greater than the square of the correlation between them.

### 2.7. Ethical Considerations

A favourable opinion was obtained from the Commission for Ethics from Administração Regional de Saúde de Lisboa e Vale do Tejo (Health for the Lisbon and Tagus Valley Regional Health Authority)—Process 086/CES/INV/2018. Ethical standards were followed regarding authorisation from the authors of the scale to translate and validate the instrument for a Portuguese population, as well as ensuring respect for human dignity, autonomy, privacy, and data confidentiality. Participants were also informed about the procedure for storing the data collected and that these would be destroyed 5 years after the end of this study.

## 3. Results

### 3.1. Exploratory Factor Analysis

Four factors were able to explain the phenomenon studied. In analysing the commonalities and variables belonging to each factor, namely factor weights under 0.40, low commonalities and correlations of >0.20 and <0.30 with other items, along with a qualitative analysis of the global contribution to the ESCA’s construct, we decided to eliminate items 1, 4, 15, 19, 25, and 30 (“I would gladly give up some of my set ways if it meant improving my health”; “I know how to get the facts I need when my health feels weakened”; “I expect to reach my peak wellness”; “If I am not good to myself, I believe I cannot be good for anyone else”; “I have a planned program for rest and exercise”; “I know what foods to eat to keep me healthy”, respectively).

After excluding these items, the factor analysis procedures were repeated. The analysis results revealed that the 29 items were organised into four factors with a total explained variance of 44% and an eigenvalue above 1. The four-factor solution is supported theoretically by the author of the instrument, which backs up the decision to extract four factors.

Bartlett’s sphericity test (5288.701, *p* = 0.000) and the Kaiser–Meyer–Olkin (KMO) measure of sampling adequacy (0.890) were considered good, showing that the factor analysis model was appropriate. The first factor explains 24.42% of the total variance, the second 8.22%, the third 6.55%, and the fourth 4.95%.

In-factor rotation was performed through Varimax rotation. The response distribution to the items on the scales (mean and standard deviation) and commonalities are presented in Table 2.

Analysing the factor structure and respective loadings of the ESCA, as shown in Table 2, we found that six items present a factor loading under 0.50, with all the others presenting higher factor loadings considered statistically significant.

### 3.2. Reliability Analysis

The reliability of the 29-item scale and the respective sub-scales was assessed through internal consistency. The Cronbach alpha for the global scale is α = 0.87, and the sub-scales are as follows: α = 0.84 for initiative and self-responsibility, α = 0.71 for self-concept, α = 0.76 for knowledge and information seeking, and α = 0.65 for passivity.

### 3.3. Confirmatory Factor Analysis

The structure obtained via EFA was submitted to CFA, with a model that showed an acceptable goodness of fit index (χ^2^/df = 3.61; CFI = 0.81; GFI = 0.87; RMSEA = 0.06; P[rmsea ≤ 0.05] < 0.001; MECVI = 2.36). The Mahalanobis distances revealed no multivariate outliers, and there were no severe violations of the normality distribution (Sk < 3 and KU < 10).

Although the model showed an acceptable goodness of fit index, there was a need to correct the trajectories in the model between the residuals of the following item pairs: e25–e28, e25–e27, e25–e26, e26–e27, e26–e28, e27–e28 (“Passivity”), e21–e24, e21–e23 (“Knowledge and Information Seeking”), e17–e19, e16–e19, e15–e19,e14–e17, e13–e16, e13–e15, e13–e14, e12–e19, e12–e17 (“self-concept”), e9–e10, e7–e8, e6–e9, e6–e8, e6–e7, e5–e11, e3–e11, e3–e10, e3–e8, e2–e7 (“Initiative and Self-responsibility”). Although the model was better adjusted, new covariances were made between e5–e8, e5–e6, e4–e5, e1–e3, and e1–e2, all belonging to the “Initiative and Self-responsibility” factor, and later between e16–e17, belonging to the “self-concept” factor. Therefore, it was possible to obtain a good quality of fit (χ^2^/df = 2.85; CFI = 0.88; GFI = 0.91; RMSEA = 0.05; P[rmsea ≤ 0.05] < 0.001; MECVI = 1.87).

Thus, the CFA revealed that the factorial model is stable and significant. The ESCA proved sufficient adjustment for use in the Portuguese cultural context.

## 4. Discussion

The cross-cultural adaptation of the ESCA for the Portuguese culture was carried out with a sample of elderly people living at home, following all the guidelines recommended for health research [21,22], which resulted in a minimal change related to its presentation: a Likert scale from complete agreement to complete disagreement, most probably related to the sociocultural context. The remaining process of analysing the psychometric properties of the Portuguese version of the scale followed the two separate processes mentioned, although partially intricated [28], aiming to ensure that the instrument maintains semantic, conceptual idiomatic, and psychometric equivalence.

An instrument consisting of 29 items was presented, 6 fewer than the original scale, and these were excluded due to their factor loadings of <0.40 and their low commonalities and correlations of >0.20 and <0.30, respectively with other items.

Important variables were considered to keep in the factor analysis are those with higher linear correlations with each other and high loadings in the component matrix before rotation and in commonalities [24].

When seeking to understand the aspects inherent to item exclusion, the only reference being the ESCA of Kearney and Fleischer successfully translated and validated in some countries, we see some coincidences among the extracted items.

Despite the cultural differences that may be found in Asian countries, we see that the item “If I am not good to myself, I believe I cannot be good for anyone else” was excluded from the Japanese [14], Turkish [15], and Cantonese versions [16]. The item “I have planned a relaxation and exercise programme” was also excluded from the Japanese version [14] as well as the item “I know how to get the facts I need when my health feels weakened” from the Cantonese version [16].

Concerning the construct validity, factors were extracted using the Varimax rotation method. Combining the analysis made using the Kaiser criterion and the scree plot criterion, the variance extracted for each factor, total explained variance, and the underlying theoretical perspective, four factors were extracted, representing 44% of the total variance, which are higher values than those of 40% obtained by the authors. Bartlett’s sphericity test (5288.701, *p* = 0.000) and the Kaiser–Meyer–Olkin (KMO = 0.890) measure showed that the factor analysis was appropriate. Considered excellent are KMO values [0.9; 1.0]; good [0.8; 0.9]; average [0.7; 0.8]; mediocre [0.6; 0.7]; bad but still acceptable [0.5; 0.6]; and ≤0.50 unacceptable. As *p* < 0.001, it is accepted that the correlation matrix is not an identity matrix [24].

This was followed by the factor naming analysis, interpreting the items and the factor-loading pattern for the items. Factor 1 is measured by Items 5, 6, 10, 11, 12, 14, 16, 17, 21, 22, and 23. Except for Items 17 and 22, belonging to the self-concept dimension, and Items 6 and 21, belonging to passivity, all others overlap the conceptually proposed initiative and self-responsibility dimension. In the analysis made, it was considered that Item 17 “I follow through on my decisions” related to individual responsibility; Item 22 “I am a good friend to myself” can be understood not as the individual’s assessment of themselves (self-esteem) but the responsibility to care for themselves. Concerning Item 6, “I tend to neglect my personal needs”, and Item 21, “I rarely carry out the resolutions I make concerning my health”, although worded negatively and with a reverse score, they corroborate aspects related to the individual’s initiative and self-responsibility, so the designation is kept.

Regarding the concept of responsibility, the International Bioethics Committee, in its report on the principle of individual responsibility as related to health [29], states that it is linked to the notions of autonomy and freedom and can be understood as the capacity to be responsible, which presupposes self-awareness, reason, aptitude to act freely, and capability of discernment as to the reach or consequences of their actions. As a result, individual responsibility is associated with the personal responsibility of seeking care for oneself and preventing the risk factors of illnesses, which are attributed to modifiable individual behaviours, so the items associated with this factor reflect aspects of individual responsibility for what should be done to take care of their health. A vision of health promotion and well-being in healthy people proposes a shared responsibility involving healthcare, education, economy, environment, and social cohesion [30], so the item “I deserve all the time and care it takes to maintain my health” reflects the person’s right to assume responsibility for health and social policies in promoting self-care.

Factor 2 is measured by Items 2, 7, 9, 27, 28, 33, 34, and 35. Except for Item 34, which was included in the sub-scale of Initiative and Self-responsibility, all the others can overlap the conceptually proposed dimension of self-concept. Item 34, “I remember when I had my last health check and will return on time for the next one”—by including in the first part the idea “I know when…” reveals aspects inherent to a person’s appreciation of themselves, referring to aspects inherent to self-confidence.

Self-concept is the set of perceptions people have about themselves, formed through interpreting their own experiences and environments and being influenced by reinforcement, feedback from significant others, and cognitive processes, such as causal attributions [31]. This concept of oneself incorporates an evaluative aspect that lets the person extract relevant information for new situations. Self-esteem, self-worth, and self-confidence are distinct but inter-related concepts and are conceptually associated with self-concept, so the designation was kept.

Factor 3 is measured using Items 18, 20, 26, 31, and 32. These items are set conceptually in the knowledge- and information-seeking dimension, so the same term is maintained. Considering the items include the phrases “I know”, “I am interested in learning”, and “I seek information” helps us to obtain a level of knowledge and look for information and aspects that appear to be associated with health literacy. According to the WHO [32], the term health literacy represents the knowledge and personal skills that accumulate through daily activities, generations, and social interactions mediated by organisational structures and the availability of resources that enable people to access, understand, appraise, and use information and services to promote and maintain good health and well-being. Reinforcing this line of thought, educational level is identified by the WHO as one of the social determinants of health, namely as intermediate social determinants. These are understood as circumstances of daily life that support good health, access to healthcare and recovery from illness [33]. This perspective theoretically reinforces the relevance of the knowledge- and information-seeking dimension in a person’s ability to engage in self-care actions.

Factor 4 is measured by Items 3, 8, 13, 24, and 29. All, except for Item 29, “I have little to contribute to others”, are set conceptually in the passivity dimension, so the nomenclature is kept.

The Cronbach alpha for the global scale (α = 0.87) and the sub-scale of initiative and self-responsibility (α = 0.84) are considered good, the sub-scales of self-concept (α = 0.71) and knowledge and information seeking (α = 0.76) as reasonable, and the sub-scale of passivity (α = 0.65) as weak but acceptable. The generally agreed-upon lower limit for Cronbach’s alpha is 0.70 [25,34], although it may decrease to 0.60 in exploratory research [34]. The values obtained in this study are similar to those found by Riesch and Hauck in 1986, whose values for the total scale and sub-scales ranged from 0.73 to 0.90.

CFA was not developed in the study by Riesch and Hauck [17], so the values obtained in this study are discussed. The four-factor model of the ESCA in the Portuguese language and culture showed an acceptable quality of fit (χ^2^/df = 2.85) since χ^2^/df values ≤ 5 are considered acceptable fit/good fit ≤ 2. The CFI value = 0.88 reveals the general adequacy of the model since values ≥ 0.90 indicate quality of fit. A considerable parsimony index was obtained (MECVI = 1.87), P[rmsea 0.05] < 0.001 for RMR values = 0.07. The GFI index = 0.91 and RMSEA = 0.05 were considered acceptable as the scores were between 0.08 and 0.05 [20,21]. The ESCA proved sufficient adjustment for use in the Portuguese cultural context.

The limitations of this study include the sample containing only elderly people living at home since different stages of life can influence the way each person understands and responds to the items in the instrument, which could affect the factor structure. We suggest future research to analyse the instrument’s psychometric properties with other populations to reinforce its reliability.

The scale presents appropriate psychometric characteristics and can be used in future research by the Portuguese scientific community and in clinical contexts. Since it can assess a person’s capacity to engage in self-care behaviour, it could guide nurses in developing strategies to promote this capacity.

## 5. Conclusions

The translation and adaptation of the ESCA to the Portuguese language and culture and the analysis of the psychometric properties presented in this study reveal an instrument comprising 29 items with suitable validity and reliability criteria to assess a person’s capacity to engage in self-care behaviour.

By contributing to providing an instrument to assess a person’s capacity to engage in self-care activities—specifically in a stage of life where vulnerability increases, either associated with the ageing process or the prevalence of complex chronic diseases, which are becoming more and more prevalent in all societies—the ESCA is an added value to identify the potential of the Portuguese individuals to involve themselves in self-care activities promoting, for example, active ageing.

Although this information is valuable not only for healthcare professionals, as self-care is a necessary ability for daily living and therefore can and should be used in different societal dimensions. It is mostly helpful for health professionals.

Integrating ESCA into healthcare will allow us to gather information and design strategies that promote greater self-concept, autonomy, health-related responsibility, and support in decision making. The promotion of self-care by health professionals emphasises the centrality of the client in healthcare and highlights the active role they can play in promoting their health and well-being.

Since the potential of an elderly person can be modified by the intervention of health professionals, mobilising this instrument before and after the intervention(s) will contribute to highlighting the effectiveness of the care provided.

Although the decision to validate this instrument in an elderly population stems from the fact that this study is part of a more comprehensive study involving elderly people, we are aware of the importance of developing the analysis of the psychometric properties of ESCA in populations with different characteristics from the Portuguese cultural context.

At the same time, the results of this study can stimulate other researchers to validate or revise this instrument in other contexts since the scope of self-care cuts across different countries and disciplinary areas. Using this instrument on others who access self-care behaviours in health/illness conditions will allow a greater understanding of the potential for self-care actions and self-care actions themselves.

## Figures and Tables

**Table 1:** General sociodemographic characteristics of the participants (N = 625).

Variables	Categories	Frequency	%
Gender	Female	420	67.2
	Male	205	32.8
Age	≥65 to 74 years	306	49
	≥75 to 84 years	255	40.8
	≥85 years	64	10.2
Residence	Live alone	152	24.3
	Partner/family/significant other	473	75.7
Education	Did not attend school	23	3.7
	Attended or completed primary school	367	58.7
	Attended or completed secondary school	189	30.2
	Higher education	46	7.4

**Table 2:** Summary descriptive statistics of the ESCA (29-item version), commonality, matrix of the items for the four-factor solution, and respective loadings.

	Item (English|Portuguese)	*h^2^*	*M*	*SD*	Factor Loadings			
					1	2	3	4
14.	I look for better ways to look after my health|Procuro melhores formas de cuidar da minha saúde.	0.57	3.24	0.88	0.649			
12.	I eat a balanced diet|Faço uma dieta equilibrada	0.42	3.16	0.92	0.627			
10	I perform certain activities to keep from getting sick|Realizo determinadas atividades para não adoecer.	0.45	3.25	0.82	0.620			
23	I take good care of myself|Cuido bem de mim.	0.49	3.06	0.96	0.574			
5	I take pride in doing the thing I need to do in order to remain healthy|Sinto orgulho em fazer aquilo que é necessário para permanecer saudável	0.41	3.41	0.82	0.569			
11	I strive to better myself|Procuro melhorar-me a mim própria	0.47	3.40	0.76	0.567			
6	I tend to neglect my personal needs|Tenho tendência a negligenciar as minhas necessidades pessoais.	0.51	2.65	1.28	0.538			
16	I deserve all the time and care it takes to maintain my health|Mereço todo o tempo e cuidado necessário para me manter saudável	0.38	3.56	0.69	0.509			
22	I am a good friend to myself|Sou um(a) bom(boa) amigo(a) de mim próprio(a).	0.43	3.29	0.90	0.497			
21	I rarely carry out the resolutions I make concerning my health|Raramente cumpro as decisões que tomo relativamente à minha saúde.	0.47	2.79	1.25	0.475			
17	I follow through on my decisions|Dou seguimento as minhas decisões	0.38	3.22	0.90	0.468			
35	I understand myself and my needs pretty well|Conheço-me bem, assim como às minhas necessidades.	0.50	3.53	0.69		0.615		
28	I take responsibility for my own actions|Sou responsável pelas minhas ações.	0.39	3.68	0.64		0.602		
2	I like myself|Gosto de mim	0.40	3.56	0.73		0.562		
9	I make my own decisions|Tomo as minhas próprias decisões.	0.32	3.49	0.74		0.519		
33	I feel I am a valuable member of my family|Sinto que sou um membro valioso da minha família.	0.31	3.62	0.71		0.511		
27	Life is a joy|A vida é uma alegria	0.27	3.15	1.03		0.509		
7	I know my strong and weak points|Conheço os meus pontos fortes e fracos	0.30	3.36	0.77		0.469		
34	I remember when I had my last health check and will return on time for the next one|Sei quando fiz o meu ultimo exame de saúde, e vou fazer o próximo no prazo previsto	0.27	3.60	0.74		0.434		
31	I am interested in learning all that I can about my body and the way it functions|Estou interessado(a) em aprender o máximo possível sobre o meu corpo e o seu funcionamento.	0.72	2.86	1.22			0.840	
26	I am interested in learning about various disease processes and how they affect me|Estou interessado(a) em aprender sobre os vários processos de doença e como estes me afetam.	0.61	3.10	1.05			0.759	
32	I seek information to care for myself|Procuro informação para cuidar de mim	0.56	2.92	1.08			0.715	
18	I have no interest in learning about my body and how it functions|Não tenho interesse em aprender sobre o meu corpo e o seu funcionamento.	0.51	2.54	1.40			0.622	
20	I understand my body and how it functions|Conheço o meu corpo e o seu funcionamento.	0.36	2.96	0.95			0.407	
13	I complain a lot about the things that bother me without doing much about them|Queixo-me muito das coisas que me incomodam, mas não faço tudo o que posso para as resolver	0.44	2.24	1.37				0.637
24	Health promotion is a chance thing for me|A promoção da saúde é uma coisa que me acontece por acaso.	0.48	2.71	1.16				0.631
3	I often feel that I lack the energy to care for needs the way I my health would like to|Costumo sentir falta de energia para cuidar das minhas necessidades de saúde da forma como gostaria	0.42	1.66	1.37				0.615
8	I often put off doing things that I know would be good for me|Costumo adiar fazer as coisas que sei que me beneficiariam	0.54	2.22	1.36				0.521
29	I have little to contribute to others|Tenho pouco a contribuir para os outros	0.43	2.90	1.16				0.517
Cronbach’s alpha coefficient					0.843	0.705	0.755	0.646

Factors: 1—initiative and self-responsibility (α = 0.843); 2—self-concept (α = 0.705); 3—knowledge and information seeking (α = 0.755); 4—passivity (α= 0.646).

## Data Availability

Data are contained within this article.
